# Intraoperative Flexible Rectoscopy for Internal Opening Identification in Transsphincteric Perianal Fistula: A Feasible and Accessible Adjunct to Seton Placement

**DOI:** 10.3390/medicina62091695

**Published:** 2026-09-04

**Authors:** Adem Aslan, Şebnem Çimen, Mahmut Baran Yerlikaya, Harun Bayram, Enes Ağırman, Rıfat Peksöz

**Affiliations:** 1 Department of General Surgery, Faculty of Medicine, Ağrı İbrahim Çeçen University, 04100 Ağrı, Türkiye; drademaslan@gmail.com (A.A.); hrnbay@hotmail.com (H.B.); 2 Department of General Surgery, Akyurt State Hospital, 06750 Ankara, Türkiye; 3 Department of General Surgery, Faculty of Medicine, Van Yüzüncü Yıl University, 65090 Van, Türkiye; gcbaranyerlikaya@gmail.com; 4 Department of General Surgery, Faculty of Medicine, Atatürk University, 25030 Erzurum, Türkiye; agirman_enes@hotmail.com (E.A.);

**Keywords:** perianal fistula, transsphincteric fistula, internal opening, flexible rectoscopy, seton, intraoperative confirmation, recurrence, anal continence

## Abstract

*Background and Objectives*: Accurate identification of the internal opening is a key determinant of success in perianal fistula surgery, as missed openings are strongly associated with persistent infection and recurrence. Although magnetic resonance imaging provides detailed preoperative mapping, intraoperative confirmation relies largely on surgical judgment. This study evaluated the technical feasibility and early observed outcomes of flexible rectoscopy-guided identification of the internal opening during loose seton placement in patients with transsphincteric perianal fistula whose internal opening could not be localized by routine assessment or pelvic MRI. *Materials and Methods*: This single-center retrospective descriptive feasibility series included 44 adult patients with cryptoglandular transsphincteric perianal fistula who underwent loose seton placement. All included patients had an internal opening that could not be localized by routine preoperative assessment or pelvic MRI. Intraoperative flexible rectoscopy was used to identify the internal opening in relation to the dentate line. The primary endpoint was successful intraoperative visualization of the internal opening. Operative time, healing time, early follow-up outcomes, Wexner continence score and patient satisfaction were descriptively recorded. *Results*: The mean age was 41.00 ± 12.06 years and the mean BMI was 29.86 ± 3.65 kg/m^2^. The internal opening was successfully visualized using flexible rectoscopy in all 44 patients (100%). Mean operative time was 6.66 ± 1.49 min and mean healing time was 59.45 ± 9.96 days. During the available 3–7-month early follow-up, no complications were documented, while recurrence occurred in one patient (2.3%) at the fourth postoperative month. The exact binomial 95% confidence interval for the observed recurrence rate was 0.06–12.02%. The median postoperative Wexner score was 0 (IQR 0–0; range 0–1). *Conclusions*: Flexible rectoscopy enabled intraoperative identification of the internal opening in all patients in this descriptive series and may represent a feasible adjunct when routine assessment and pelvic MRI fail to localize the opening. The absence of a comparator and the relatively short follow-up prevent conclusions regarding comparative accuracy, safety or recurrence reduction. Larger prospective comparative studies with long-term follow-up are required.

## 1. Introduction

Perianal fistulas are chronic inflammatory anorectal disorders characterized by the formation of an epithelialized tract between the anal canal and the perianal skin, with a high potential for recurrence. The majority of cases develop secondary to cryptoglandular infection and the Parks classification continues to serve as the fundamental reference for defining fistula anatomy and guiding surgical strategy [1]. Particularly, in transsphincteric and other complex fistula types, the surgical approach requires a delicate balance between eradication of the disease and preservation of anal continence.

The most critical determinant of success in fistula surgery is accurate identification of the internal opening and intervention along the correct anatomical tract [2]. Failure to identify the internal opening is directly associated with persistent infection, secondary tract formation and recurrence [3]. In complex fistulas, preoperative magnetic resonance imaging (MRI) is recommended due to its high diagnostic accuracy and plays an important role in surgical planning [4,5]. However, MRI does not provide intraoperative guidance and does not allow real-time confirmation of the internal opening.

Seton placement is widely used as a sphincter-preserving approach, particularly in transsphincteric and high fistulas [6]. Nevertheless, insufficient confirmation of the anatomical accuracy of the internal opening during seton placement may reduce surgical success and lead to recurrence. Video-assisted anal fistula treatment (VAAFT) and laser-based techniques enable direct visual control of the internal opening; however, they require advanced technology, additional costs and specialized equipment [7,8].

Rectoscopy is a widely accessible and low-cost method that allows direct evaluation of the anal canal and distal rectum [9,10]. Although it is commonly used as part of clinical examination, its role in intraoperative confirmation of the internal opening and its contribution to surgical outcomes have not been systematically evaluated. In particular, the ability of flexible rectoscopy to allow simultaneous visual confirmation of the internal opening via its working channel may provide a potential advantage in improving anatomical accuracy.

In modern fistula surgery, not only anatomical closure but also long-term disease control and preservation of anal continence are considered key measures of success [3,5]. Given that accurate localization of the internal opening is closely associated with surgical outcomes [2,3], the impact of intraoperative direct visualization on the surgical accuracy chain and patient-centered outcomes warrants re-evaluation.

This study aimed to describe the technical feasibility of flexible rectoscopy-guided identification of the internal opening during loose seton placement in patients with transsphincteric perianal fistula whose internal opening could not be localized by routine assessment or pelvic MRI. Secondary objectives were to report operative characteristics and early observed clinical and functional outcomes.

## 2. Materials and Methods

### 2.1. Study Design and Ethical Approval

This study was conducted as a single-center, retrospective, descriptive feasibility series. The study protocol was approved by the Scientific Research Ethics Committee of Ağrı İbrahim Çeçen University on 27 November 2025 (Decision No: 518). The study was performed in accordance with the principles of the Declaration of Helsinki. All patient data were anonymized prior to analysis and used solely for scientific purposes.

### 2.2. Study Setting and Patient Selection

The study was carried out using records from the General Surgery Clinic and the Gastroenterology Endoscopy Unit of Ağrı Training and Research Hospital. Consecutive patients treated between 1 January 2026 and 15 May 2026 were assessed for eligibility.

Patients aged 18 years and older with cryptoglandular transsphincteric perianal fistula were eligible if the internal opening could not be localized by routine clinical assessment or preoperative pelvic MRI and flexible rectoscopy-guided loose seton placement was subsequently performed. Fistulas associated with malignancy, Crohn’s disease, traumatic or obstetric causes, as well as patients with incomplete clinical or follow-up data, were excluded from the analysis.

**Figure 1:**
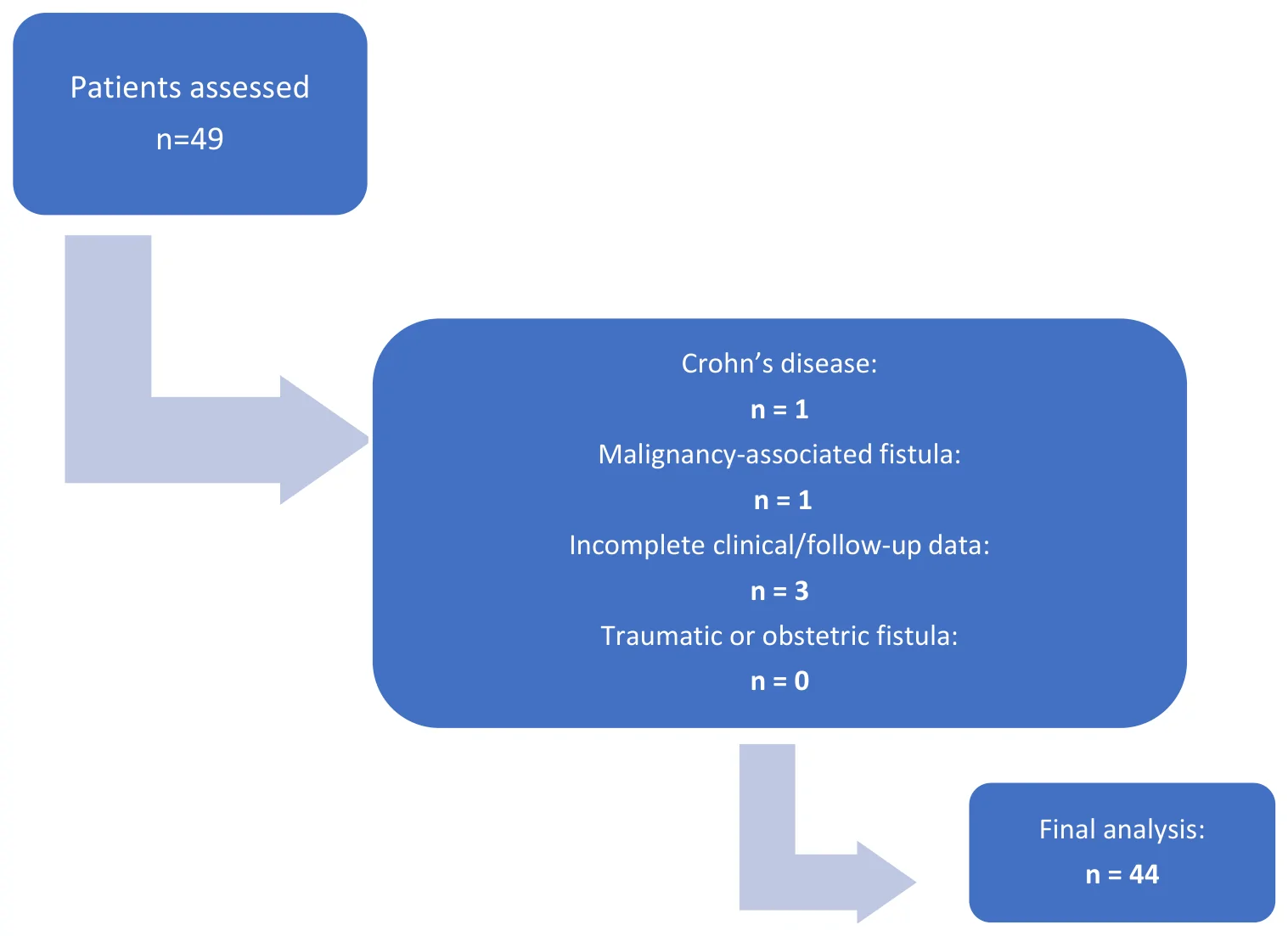
Patient-selection flow diagram.

During the study period, 49 patients were assessed for eligibility. Five patients were excluded: one because of Crohn’s disease, one because of malignancy-associated fistula and three because of incomplete clinical or follow-up data. No patient was excluded because of a traumatic or obstetric fistula. The remaining 44 patients constituted the final study cohort (Figure 1).

### 2.3. Preoperative Assessment

All patients underwent detailed anamnesis and physical examination prior to the procedure. Fistula anatomy was classified according to the Parks classification system. External opening localization, number of external openings and presence of secondary tracts were recorded.

All included patients underwent non-contrast pelvic MRI for preoperative mapping of the fistula anatomy. The MRI protocol included T2-weighted and fat-suppressed sequences, with imaging planes oriented to assess the anal canal, fistula trajectory, and its relationship with the sphincter complex. MRI examinations were interpreted as part of routine clinical practice by radiologists with approximately 5–20 years of experience. The examinations were not systematically reviewed by a dedicated pelvic radiologist, and a dedicated structured reporting template for perianal fistula was not routinely used. Importantly, inability to reliably localize the internal opening by routine clinical assessment or preoperative pelvic MRI was an inclusion criterion of the present study. Therefore, the absence of MRI localization of the internal opening in all 44 included patients reflects the selected nature of the study cohort and should not be interpreted as the diagnostic failure rate of MRI in the overall perianal fistula population. Tract length was recorded according to intraoperative measurements and imaging findings.

### 2.4. Surgical Procedure

All procedures were performed under general anesthesia. Patients were placed in the left lateral decubitus position with the hips and knees flexed. Rectal preparation consisted of one enema on the evening before the procedure and another on the morning of the procedure. This represented the routine institutional perioperative practice during the study period; routine antibiotic prophylaxis and tract irrigation were not performed.

Flexible rectoscopy was performed using a standard flexible endoscope introduced to visualize the anal canal and distal rectum, with the dentate line used as the principal anatomical reference. Flexible endoscopic forceps were gently advanced through the fistula tract using tactile feedback. The instrument was not advanced forcibly and progression was discontinued if resistance was encountered to minimize the risk of perforating the fistula wall or creating a false passage. A second endoscopic forceps was introduced through the working channel of the rectoscope. Endoluminal visualization and approximation of the forceps tips were used to identify the internal opening and confirm continuity with the external fistula tract. No additional secondary tract was documented during preoperative assessment, MRI evaluation, or intraoperative exploration. All patients had a single external opening. Previous fistula surgery was not an exclusion criterion; one patient had undergone one previous fistula operation. Horseshoe extension, supralevator extension, and abscess cavities were not captured as separate predefined variables in the retrospective dataset.

A number 1 polypropylene suture (Prolene^®^ Ethicon Inc., Cincinnati, OH, USA) was used as the seton material. The suture was passed through the tract under controlled guidance via the forceps and positioned along the anatomical tract as a loose seton.

Loose seton placement was performed in all patients. Operative time was systematically recorded in minutes and defined as the interval from insertion of the flexible rectoscope to completion of loose seton placement. Patient positioning, induction of general anesthesia, procedural preparation and recovery time were not included. The key steps of rectoscopy-guided internal opening localization and seton placement technique were presented as Supplementary Materials Video S1. 

### 2.5. Data Variables

Demographic variables included age, sex, body mass index and American Society of Anesthesiologists (ASA) score. All patients included in the study had transsphincteric-type perianal fistula. Clinical variables included tract length, number of external openings and pelvic MRI findings. Surgical variables included the method of internal opening identification and operative time. Technical variables included successful visualization of the internal opening, concordance with preoperative localization, identification of secondary tracts and completion of loose seton placement through the visualized tract.

Postoperative assessment included complications documented during the available early follow-up, healing time, follow-up duration and recurrence observed during the available follow-up period. Anal continence was evaluated using the Wexner continence score. The Wexner continence score was recorded on postoperative day 15 and was reported descriptively as median, IQR, and range. No standardized preoperative Wexner score was available; therefore, quantitative change from baseline could not be assessed. Overall treatment satisfaction was assessed on postoperative day 15 using a single, non-validated five-point Likert question (1 = very dissatisfied; 5 = very satisfied). The assessment was performed during routine clinical follow-up and did not include an anonymous or multidimensional validated patient-reported outcome measure.

### 2.6. Definitions

Recurrence was defined as the reappearance of a fistula tract in the same anatomical region after clinical healing had been achieved and confirmed by physical examination. Complications were defined as adverse clinical events documented during the available 3–7-month early follow-up period. Healing was defined as complete epithelialization of the surgical site accompanied by the absence of persistent fistulous discharge, clinically evident persistent tract or abscess, and no requirement for additional fistula-related intervention. Healing time was defined as the interval from the procedure to fulfillment of these clinical criteria.

### 2.7. Outcome Measures

The primary endpoint was successful intraoperative visualization and identification of the internal opening using flexible rectoscopy. Secondary descriptive endpoints included completion of loose seton placement, operative time, healing time, complications documented during the available early follow-up, recurrence observed during the available follow-up, postoperative continence status and overall treatment satisfaction.

### 2.8. Statistical Analysis

Statistical analyses were performed using IBM SPSS Statistics (version 29.0; IBM Corp., Armonk, NY, USA). Given descriptive study design and absence of a comparator group, no hypothesis-testing, correlation or multivariable analyses were performed. Continuous variables were summarized using mean ± standard deviation or median and interquartile range, as appropriate. Categorical variables were reported as numbers and percentages. An exact binomial 95% confidence interval was calculated for the observed recurrence proportion to quantify the uncertainty surrounding the recurrence estimate.

## 3. Results

A total of 44 patients were included in the study. The mean age was 41.00 ± 12.06 years and the mean body mass index was 29.86 ± 3.65 kg/m^2^. All cases had a transsphincteric-type perianal fistula and loose seton was applied in all patients. Demographic and baseline clinical characteristics are presented in Table 1.

All patients had a single external opening. External openings were located at the 2-, 3-, 4-, 5-, 6-, 7-, and 9-o’clock positions in 1 (2.3%), 8 (18.2%), 1 (2.3%), 7 (15.9%), 15 (34.1%), 10 (22.7%), and 2 (4.5%) patients, respectively. The internal openings identified intraoperatively were located at the 3-, 4-, 5-, 6-, and 7-o’clock positions in 11 (25.0%), 7 (15.9%), 7 (15.9%), 15 (34.1%), and 4 (9.1%) patients, respectively. Mean fistula tract length was 3.68 ± 1.01 cm (range, 2–6 cm). Forty-three patients (97.7%) had no history of previous fistula surgery, whereas one patient (2.3%) had undergone one previous fistula operation. High versus low transsphincteric classification, exact distance from the anal verge, and prior abscess drainage were not available as separately recorded variables in the retrospective dataset.

Flexible rectoscopy enabled successful visualization and identification of the internal opening in all 44 patients, corresponding to a technical success rate of 100%. Because the internal opening had not been localized before the procedure, rectoscopy did not change a previously established location but provided intraoperative identification in all included cases. No secondary tracts were identified and loose seton placement through the identified tract was successfully completed in all patients.

The mean operative time was 6.66 ± 1.49 min. The mean healing time was 59.45 ± 9.96 days. Operative and early clinical outcomes are summarized in Table 1 and Table 2.

The available follow-up duration ranged from 3 to 7 months, with a mean duration of 5.00 ± 1.06 months. No complications were documented during this early follow-up period. Recurrence occurred in one patient (2.3%) at the fourth postoperative month.

The exact binomial 95% confidence interval for the observed recurrence proportion was 0.06–12.02%.

On postoperative day 15, the median Wexner continence score was 0 (IQR 0–0; range 0–1). One patient had a Wexner score of 1. Overall treatment satisfaction, assessed on postoperative day 15 using a single non-validated five-point Likert question, had a median score of 4 (IQR 4–5; range 3–5). Detailed descriptive statistics for the functional outcomes are presented in Table 1.

## 4. Discussion

One of the principal determinants of success in perianal fistula surgery is accurate localization of the internal opening and intervention along the correct anatomical tract. Failure to identify the internal opening is regarded as a fundamental mechanism that significantly increases the risk of persistent infection, secondary tract formation and recurrence. A recent review addressing the management of recurrent anal fistulas emphasized that failure to correctly identify the internal opening is strongly associated with recurrence and that intraoperative confirmation represents a critical step; indeed, missing the internal opening has been reported to increase the likelihood of recurrence by up to twentyfold [11]. Furthermore, in complex cryptoglandular fistulas, situations in which the internal opening cannot be identified despite clinical examination, examination under anesthesia or even detailed magnetic resonance imaging are associated with higher recurrence rates, and management of this subgroup is particularly challenging [11,12]. In this context, integrating flexible rectoscopy into the seton placement procedure as a real-time visual identification method may represent a rational, accessible and low-cost approach.

In the present descriptive feasibility series, flexible rectoscopy enabled intraoperative visualization and identification of the internal opening with reference to the dentate line in all 44 patients with transsphincteric cryptoglandular perianal fistula. In all included patients, the internal opening had not been localized by routine preoperative assessment or pelvic MRI. Therefore, flexible rectoscopy did not change a previously established localization but provided intraoperative identification in cases in which previous localization had been unsuccessful. No complications were documented during the available 3–7-month follow-up, while recurrence occurred in one patient (2.3%) at the fourth postoperative month. However, these findings represent early postoperative observations and should not be interpreted as evidence of established safety or long-term recurrence control. The exact binomial 95% confidence interval for the observed recurrence proportion ranged from 0.06% to 12.02%, indicating substantial uncertainty around the recurrence estimate despite the low observed event rate. Recurrence rates following anal fistula surgery vary widely in the literature; for example, a contemporary retrospective analysis reported an overall recurrence rate of 12.5% after definitive repair, with higher rates in complex cases, while other long-term series have demonstrated combined persistent and recurrent disease rates approaching twenty percent [13,14]. Therefore, maintaining the balance between recurrence control and preservation of continence remains the central objective of modern fistula surgery.

Preoperative imaging, particularly pelvic magnetic resonance imaging, is widely recommended in complex fistulas because of its high diagnostic accuracy in tract mapping and internal opening localization. Halligan and colleagues reported that magnetic resonance imaging may be more accurate than surgical assessment and that MRI-guided surgery may reduce recurrence by approximately seventy-five percent [15]. However, magnetic resonance imaging does not provide real-time intraoperative confirmation, and final identification of the internal opening during surgery still depends on intraoperative findings and surgical judgment. In the present cohort, pelvic MRI provided information regarding the fistula trajectory and its relationship with the sphincter complex but did not permit reliable localization of the internal opening. The approach described in this study may therefore be considered a practical complementary strategy that adds direct intraoperative identification to preoperative anatomical mapping. Consensus documents standardizing the role of magnetic resonance imaging and other imaging modalities have also emphasized that accurate classification and mapping, particularly in complex disease, are closely associated with clinical outcomes [16,17].

In recent years, endoscopic and fistuloscopy-based techniques such as VAAFT have attracted attention because they enable direct visualization of the internal opening. However, long-term data indicate that success rates vary according to patient selection and technical factors. A 2023 long-term series reported recurrence rates increasing from 29% at one year to 63% at five years [18]. A 2022 meta-analysis concluded that heterogeneity and variable follow-up durations preclude definitive conclusions regarding the overall effectiveness and safety of VAAFT [19]. Moreover, advanced techniques such as VAAFT require dedicated equipment and additional costs, which may limit their availability across centers. In contrast, flexible rectoscopy is widely available in most institutions and may provide a lower-barrier alternative for intraoperative identification of the internal opening. Because this approach uses standard flexible endoscopy equipment that is already available in many institutions, it may be particularly accessible in resource-limited settings. However, no formal cost or cost-effectiveness analysis was performed; therefore, an economic advantage cannot be concluded from the present findings.

Loose seton placement is a long-established sphincter-preserving strategy and is not a novel component of the present technique. The potentially distinctive aspect of the described approach is the combination of loose seton placement with flexible rectoscopy to identify the internal opening intraoperatively when routine assessment and pelvic MRI have been inconclusive. Its principal advantage lies in maintaining drainage and controlling sepsis without compromising the sphincter complex. Nevertheless, passage of the seton through an incorrect or false tract may predispose to treatment failure and morbidity, highlighting the importance of accurate internal opening identification. The type and handling of the instrument used for tract catheterization are also important because forceful advancement of a rigid instrument through a deep or tortuous tract may perforate the fistula wall and create a false passage. Curved artery forceps may facilitate controlled catheterization when conventional probing is required. In the present series, biopsy forceps were advanced gently using tactile feedback, without forceful progression, and advancement was discontinued whenever resistance was encountered. Final identification of the internal opening was achieved under direct endoluminal visualization. In a staged drainage seton series, the overall recurrence rate was 6.5%, rising to 57.1% in patients with horseshoe extension, while incontinence was reported in 3.8% of cases [20]. In studies evaluating draining seton as definitive management, recurrence rates after seton removal have been reported in single-digit percentages, such as 7.1% [21]. In our study, the median postoperative Wexner score was 0, and one patient had a score of 1. Nevertheless, the absence of a preoperative Wexner assessment and the early postoperative evaluation prevent conclusions regarding changes in continence or long-term functional safety. Similar outcomes have been reported in selected sphincter-preserving strategies following seton placement, although results vary depending on patient selection and duration of follow-up [22].

The available follow-up in the present study ranged from only 3 to 7 months. Long-term series have demonstrated that the majority of recurrences occur within the first twenty-four months after surgery [13]. Therefore, the available follow-up was inadequate for evaluating long-term recurrence or delayed complications. Furthermore, the absence of documented complications in a cohort of 44 patients does not exclude uncommon events such as delayed abscess formation, persistent drainage, pain, continence deterioration or the need for reoperation. Standardized follow-up extending for at least 12–24 months will be necessary in future studies.

Several clinically relevant questions remain unanswered and should be addressed in future studies. Specifically, comparative studies are needed to determine whether flexible rectoscopy can identify internal openings that remain undetected during expert examination under anesthesia, whether direct visualization reduces false tract placement, and whether this translates into lower recurrence after seton removal or subsequent definitive repair. Future studies should also determine which fistula anatomies are most likely to benefit from this approach and evaluate its reproducibility, operator dependency, and feasibility when performed by surgeons without gastroenterology support. These questions cannot be answered by the present descriptive series and require prospective comparative evaluation.

This study has several important limitations. It represents a retrospective, single-center descriptive feasibility series of 44 patients and includes no conventional seton or VAAFT comparator. The highly selected cohort consisted exclusively of patients whose internal opening could not be identified by routine assessment or pelvic MRI. Follow-up was limited to 3–7 months and was inadequate for evaluating long-term recurrence or delayed complications. Continence and satisfaction were assessed early, no preoperative Wexner score was available for comparison, and satisfaction was measured using a single non-validated question. Furthermore, no formal evaluation of cost-effectiveness was performed. Therefore, the findings should be interpreted only as preliminary evidence of technical feasibility rather than evidence of comparative clinical benefit.

In summary, flexible rectoscopy enabled intraoperative identification of the internal opening and completion of loose seton placement in all 44 patients in this descriptive series. The technique may represent an accessible and time-efficient intraoperative adjunct when routine assessment and pelvic MRI fail to localize the internal opening. However, the absence of a comparator and the short follow-up prevent conclusions regarding comparative accuracy, established safety, continence preservation or recurrence reduction. Prospective comparative studies evaluating rectoscopy-guided versus conventional seton placement, with stratification by magnetic resonance imaging findings and secondary tract presence and with follow-up of at least twelve to twenty-four months, are required to determine the clinical value of this approach [13,14].

## 5. Conclusions

Flexible rectoscopy enabled intraoperative identification of the internal opening and completion of loose seton placement in all 44 patients in this descriptive feasibility series. The technique may represent an accessible intraoperative adjunct when routine assessment and pelvic MRI fail to localize the internal opening. However, the absence of a comparator and the 3–7-month follow-up prevent conclusions regarding comparative accuracy, established safety, continence preservation or recurrence reduction. Larger prospective comparative studies with long-term follow-up are required.

## Figures and Tables

**Table 1:** Descriptive characteristics and operative outcomes of the study cohort (*n* = 44).

Variable	Descriptive Value	Min–Max
Age	41.00 ± 12.06	19–68
BMI	29.86 ± 3.65	23.5–36.7
Operative time (min)	6.66 ± 1.49	5–12
Healing time (days)	59.45 ± 9.96	38–75
Follow-up duration (months)	5.00 ± 1.06	3–7
Wexner score ^1^	0 (0–0)	0–1
Satisfaction (1–5) ^2^	4 (4–5)	3–5

Continuous variables are presented as mean ± standard deviation, except for the Wexner continence score and satisfaction score, which are presented as median (interquartile range). ^1^ The Wexner continence score was assessed on postoperative day 15 and ranges from 0 to 20, with higher scores indicating worse continence. ^2^ Overall treatment satisfaction was assessed on postoperative day 15 using a single non-validated five-point Likert question (1 = very dissatisfied; 5 = very satisfied).

**Table 2:** Clinical outcomes and early postoperative results (*n* = 44).

Outcome	*n* (%)
Transsphincteric fistula	44 (100%)
Internal opening not localized by preoperative assessment or pelvic MRI	44 (100%)
Successful internal opening visualization using flexible rectoscopy	44 (100%)
Successful loose seton placement through the identified tract	44 (100%)
Secondary tract identified	0 (0%)
Complication during the available early follow-up ^1^	0 (0%)
Recurrence during the available early follow-up ^2^	1 (2.3%)
Clinically documented deterioration in continence	1 (2.3%)

Exact binomial 95% confidence interval for the observed recurrence proportion: 0.06–12.02%. ^1^ Complications were assessed during the available 3–7-month early follow-up period. The absence of documented complications should not be interpreted as excluding uncommon or delayed adverse events. ^2^ Recurrence was defined as the reappearance of a fistula tract in the same anatomical region after documented clinical healing, confirmed by physical examination during follow-up. Recurrence occurred in one patient at the fourth postoperative month. Because follow-up was limited to 3–7 months, this outcome represents only an early postoperative observation.

## Data Availability

The datasets generated and/or analyzed during the current study are available from the corresponding author on reasonable request.

## Supplementary Materials

The following supporting information can be downloaded at https://www.mdpi.com/article/10.3390/medicina62091695/s1, Video S1: Intraoperative view demonstrating the surgical procedure and the key operative steps described in the manuscript.
